# Notes and News

**Published:** 1900-11-17

**Authors:** 


### NOTES AND NEWS.

THE Red Cross Society has now withdrawn its services from Natal. The Princess of Wales' hospital ship is preparing to come home.

SIR WILLIAM THOMSON, M.D., has been appointed Surgeon-in-Ordinary to the Queen, in succession to the late Sir William Stokes.

THE Metropolitan Ear, Nose, and Throat Hospital, in Grafton Street, has received a legacy of £30,000 from the late Mr. Richard Hovis Smith. In consequence of this the institution will be altered and extended.

THE Indian Civil Medical Service is beginning to feel the strain of the continual inroads into its services made by the military authorities. Ever since 1895, the Civil Medical Service has been depleted for military requirements, and localities have consequently suffered. It is now proposed to strengthen the medical service, as it appears likely that military operations will continue to require additional medical service.

IT is a curious thing, and one of which it is not easy to find an explanation, that not only has the birth-rate seriously diminished in England, but that even our dependencies are suffering from the same strange deficiency in fecundity. The report of the Sanitary Commissioner in India shows that for 1898, the last year for which information is available, in every province except the North-Western Provinces and Oudh, the Central Provinces and Lower Burma, the birth-rates recorded were lower than those in the preceding year. It is to be noted, however, that at the same time the death-rate was much less than in the preceding year.

AT an inquest held recently at Westminster on a woman who died from the effects of an illegal operation alleged to have been performed by a Mrs. Katz, otherwise "Mrs. Evans," Mr. J. Troutbeck, the coroner, made some strong remarks about the manner in which a certain class of newspapers accepted advertisements of the kind referred to in this case, and said that a large number of the cases such as they were investigating that day were distinctly traceable to such advertisements. The jury returned a verdict of wilful murder against Katz, and added a rider to the effect that greater care should be exercised in newspaper offices in the acceptance of midwifery advertisements.

THE second expedition sent out by the Liverpool School of Tropical Medicine to investigate malarial fever has just returned from West Africa, well satisfied with their experience.

THE Duke of Portland opened the new operating theatre at the Royal Infirmary, Sheffield, last week. There yet remains a sum of nearly £12,000 necessary to defray the cost of the new institution.

MR. DHANJIBHOY, C.I.E., of Rawal Pindi, India, has further shown his goodwill to the British Government by forwarding fifty fully-equipped ambulance tongas to China for the use of the English troops.

PROTESTS are being received by the Local Government Board against certain drainage by-laws proposed by the London County Council. The objection raised is against the increase in the number of drainage pipes, which will add to cost of construction.

THERE is an epidemic of smallpox at present in Paris, but it is hoped that it is now on the wane. It is not confined to any particular district, and some uneasiness is felt by the authorities. It is reported that in one street alone in the 18th Arrondissement 800 persons have been vaccinated.

MR. J. R. TURLE has given £5,000 in aid of the building fund of the School of Medicine for Women at the Royal Free Hospital. The school is affiliated to the University of London. The new buildings are estimated to cost £35,000. They are now completed, though a debt of £8,000 still remains upon them.

PROFESSOR HITZIG of Halle will deliver the Hughlings Jackson lecture before the Neurological Society on November 29th at 9 o'clock, in the theatre of the Examination Hall, Victoria Embankment. The lecture, which will be given in English, is entitled "Hughlings Jackson and the Cortical Motor Centres in the light of Physiological Research," and is open to all medical men.

SOME time ago we referred to the proposal to introduce Chinese labour into our laundries, and commented upon the undesirability of the proceeding from a hygienic point of view. The movement is meeting with objection from the society for the employment of women. A resolution was passed at a meeting which included the opinion that the introduction of Chinese elsewhere has been found "a serious menace to the health of the State."

THE new wing at the Royal Boscombe and West Hants Hospital, which was opened last week, consists of two new wards with ward kitchens and offices, and one of the isolation wards and adjoining lavatories has been made into a temporary operating room. The new wards are connected with old building by temporary corridor, the old building being used as Administrative Block and Outpatients' Departments. The beds will be twenty, and four cots. The flooring is of terrazzo, and walls and ceilings are painted. There are no sharp angles, electric light is used, and Doulton's patent stoves in the wards, and smoke and air flues through floors have been adopted. The corridors are of white brick to dado height, then painted walls. The cost was £7,900.

